# Multiband RF pulse design for realistic gradient performance

**DOI:** 10.1002/mrm.27411

**Published:** 2018-09-14

**Authors:** Samy Abo Seada, Anthony N. Price, Torben Schneider, Joseph V. Hajnal, Shaihan J. Malik

**Affiliations:** ^1^ School of Biomedical Engineering & Imaging Sciences King’s College London, King’s Health Partners, St Thomas’ Hospital London SE1 7EH; ^2^ Philips UK Guildford United Kingdom

**Keywords:** gradient frequency characterization, minimum duration, multiband, RF pulse design, time optimal, VERSE

## Abstract

**Purpose:**

Simultaneous multi‐slice techniques are reliant on multiband RF pulses, for which conventional design strategies result in long pulse durations, lengthening echo‐times so lowering SNR for spin‐echo imaging, and lengthening repetition times for gradient echo sequences. Pulse durations can be reduced with advanced RF pulse design methods that use time‐variable selection gradients. However, the ability of gradient systems to reproduce fast switching pulses is often limited and can lead to image artifacts when ignored. We propose a time‐efficient pulse design method that inherently produces gradient waveforms with lower temporal bandwidth.

**Methods:**

Efficient multiband RF pulses with time‐variable gradients were designed using time‐optimal VERSE. Using VERSE directly on multiband pulses leads to gradient waveforms with high temporal bandwidth, whereas VERSE applied first to singleband RF pulses and then modulated to make them multiband, significantly reduces this. The relative performance of these approaches was compared using simulation and experimental measurements.

**Results:**

Applying VERSE before multiband modulation was successful at removing out‐of‐band slice distortion. This effectively removes the need for high frequency modulation in the gradient waveform while preserving the benefit of time‐efficiency inherited from VERSE.

**Conclusion:**

We propose a time‐efficient RF pulse design that produces gradient pulses with lower temporal bandwidth, reducing image artifacts associated with finite temporal bandwidth of gradient systems.

## 1 INTRODUCTION

Simultaneous multi‐slice (SMS) imaging uses multiband (MB) RF pulses to accelerate MR image acquisition by acquiring data from multiple slices simultaneously.^1^, ^2^ A simple method for designing an MB pulse is to multiply a singleband (SB) pulse by a modulation function that replicates the slices in the frequency domain.^1^, ^3^ This method quickly reaches hardware limits on peak amplitudes as the number of slices increases, forcing pulse designers to either increase the pulse duration or reduce the flip angle, both of which are problematic for sequences such as spin‐echo diffusion imaging and turbo spin echo (TSE) where high signal and short echo times are important.^4^, ^5^, ^6^ Similarly, specific absorption rate (SAR) constraints in MB SSFP applications force the use of sub‐peak amplitude MB RF pulses, which have long pulse durations and become difficult to fit within TR constraints.^7^, ^8^

A range of solutions have been proposed to reduce the peak amplitude of MB waveforms including phase‐optimization,^9^, ^10^, ^11^, ^12^, ^13^ time‐shifting,^10^, ^14^ and root‐flipping.^15^ These methods aim to reduce the peak amplitude for a given constant slice selection gradient. Alternatively, “power independent of number of slices” (PINS)^16^ pulses use a different paradigm in which an SB waveform is split into discrete subpulses and undersampled to create a periodic excitation in the slice‐select direction. This method has low RF energy but generally long pulse durations, especially for designs with large slice‐gaps. It can be made more efficient (either in time or RF energy) in combination with more traditional MB pulses—this method is known as MultiPINS.^17^

PINS pulses do not use a constant selection gradient, rather the gradient is switched on and off periodically. Taking this further, there has been recent interest in designing combinations of RF pulses and time‐variable selection gradients, which together yield the minimum possible duration. Such pulses were designed for the ISMRM pulse design challenge in 2016^18^ where participants used time‐optimal VERSE algorithms^19^, ^20^, ^21^ and the winning technique used an algorithm that designed RF pulses using an optimal control approach.^22^, ^23^, ^24^ These solutions are typically associated with very fast temporal modulation of both RF and gradient waveforms, which can be problematic if the temporal output bandwidth of the RF and gradient systems is not sufficient. In practice, however, the output bandwidth for RF chains far exceeds that of a gradient system, which implies that fast switching gradient waveforms are unlikely to be reproduced with high fidelity.

Recent work has demonstrated that for both RF pulse design^25^ and image reconstruction,^26^ limited temporal bandwidth of commercial MRI gradient systems leads to errors when gradient waveforms with high temporal bandwidth are demanded. The effective bandwidth of a gradient system relates to eddy currents,^27^, ^28^ but also to the design of the gradient coil and amplifier bandwidth.^19^ Performance can vary between manufacturers, different models and types (body vs. head), and also different orientations. Under the assumption that the system is linear time invariant (LTI), however, all of these factors can be captured by measuring the gradient impulse response function (GIRF)^26^ for any particular system.

Although hardware limits such as peak slew rate and amplitudes can be enforced as static constraints, it is not straightforward to directly incorporate a GIRF into a time‐optimal design as temporal bandwidth is a function of the complete waveform. The result is that such pulses are prone to gradient distortion related artifacts, as will be demonstrated later.

**Figure 1:**
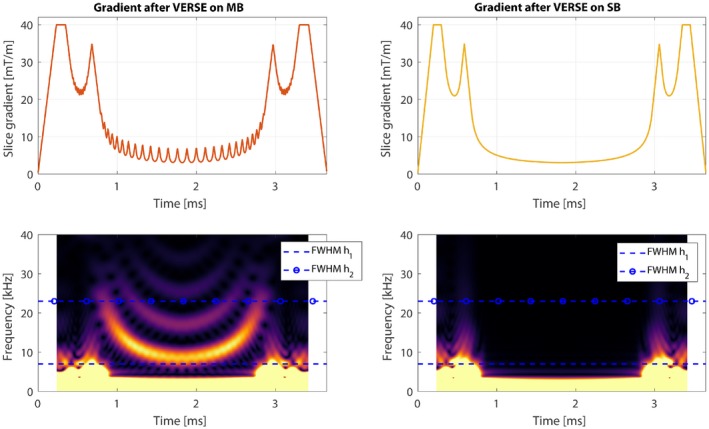
A time‐variable gradient as a result of VERSE, when applied to a multiband (MB) pulse (left column) and a singleband (SB) pulse (right column), both stretched to have matched durations. The gradient pulses have similar shapes, but the left pulse contains additional high‐frequency components, which the VERSE method translates from a highly‐modulated MB RF pulse. The bottom row shows spectrograms with 2 dotted lines marking the FWHM of a measured and duplicated gradient impulse response function (GIRF), h_1_ and h_2_, respectively. Any high‐frequency (HF) components from beyond the FWHM will be attenuated because of the GIRF. These HF components are not present in the right pulse, leading to reduced slice profile distortions

The focus of this work was to produce time‐optimal MB designs that avoid very high bandwidth demands on the gradient system. As shown in Hargreaves et al.,^19^ when VERSE is applied on SB gradients, the gradient pulses retain manageable bandwidth demands. With this in mind, we combined multiband pulses and VERSE in 2 different ways and compared their associated gradient waveforms. Firstly, the time‐optimal VERSE method^20^, ^29^ was applied directly on an MB pulse. Secondly, we applied VERSE first to an SB pulse, before applying MB modulation (which alters the RF pulse and leaves the VERSE gradient pulse intact). We hypothesized that the latter approach would benefit from a gradient waveform with lower temporal bandwidth and therefore suffer less from slice profile distortions. This concept is shown in Figure 1.

In this work, we investigate the slice profile effect because of imperfect gradients on time‐optimal MB pulses by using VERSE (for both linear and non‐linear phase pulses), PINS, and MultiPINS. We report on slice profile error, pulse durations, RF energy, and off‐resonance effects. We show that optimizing a time‐variable gradient for a SB waveform before MB modulation produces short duration RF pulses, while effectively reducing slice profile errors, and demonstrate this experimentally.

## 2 THEORY

The time‐optimal VERSE approach as applied to RF pulse design is described in Lee et al.^20^ and is referred to as VERSE in this work. For a given combination of RF and gradient pulses, it returns revised versions of these that minimize transmit time, subject to peak B_1_ (B_1,max_), gradient amplitude (G_max_) and slew rate (S_max_) constraints. We consider 2 approaches to combining this with MB RF pulse design: (1) design an MB pulse for a constant gradient, then apply the VERSE algorithm (MBv); and (2) design an SB pulse for a constant gradient, apply VERSE, then modulate it to form an MB pulse (vMB).

For the first approach phase‐optimized^11^ MB RF pulses were designed, which were then optimized using VERSE. The second approach applies VERSE to a standard SB pulse before applying a modulation function to produce an MB pulse. For a constant gradient MB pulse, exciting N slices, this function is defined as

(1) $$f_{N}\left(t\right)=\sum_{n=1}^{N}e^{i\left(\gamma Gtx_{n}+\phi_{n}\right)}$$

where γ is the gyromagnetic ratio, G is the amplitude of the constant selection gradient, t is a time‐variable, $x_{n}$ is the spatial location of the n^th^ excited slice and $\phi_{n}$ is the phase‐offset of this slice, numerically optimized as in Wong^11^ and Abo Seada et al.^30^ After application of VERSE the gradient waveform is time‐variable, and this must be accounted for in the modulation function $f_{N}^{v}\left(t\right)$

(2) $$f_{N}^{v}\left(t\right)=\sum_{n=1}^{N}e^{i\left(k\left(t\right)x_{n}+\phi_{n}\right)}$$

where the spatial frequency variable k(t) is defined as

(3) $$k\left(t\right)=-\gamma \int_{t}^{T}G\left(s\right)ds$$

When using the vMB method (i.e., performing VERSE on an SB pulse) the B_1,max_ constraint must be reduced to account for the fact that after MB modulation the amplitude will be increased. In other words, for vMB, the B_1,max_ amplitude constraint for exciting N slices becomes:

(4) $$B_{1,max,N}^{\mathrm{SB}}=B_{1,max}\max\left\{\left|f_{N}^{v}\left(t\right)\right|\right\}$$

Constant gradient MB pulse design methods that use non‐linear through‐slice phase patterns enhance performance when this phase dispersion is acceptable. To design MBv pulses with non‐linear through‐slice phase, we applied VERSE to MB pulses designed using the root‐flipping method.^15^ We refer to this method as non‐linear MBv. Furthermore, we can design vMB pulses of this kind by applying VERSE to a non‐linear SB waveform (in our case, quadratic phase)^31^ and then apply modulation function $f_{N}^{v}\left(t\right)$. We refer to this method as non‐linear vMB.

## 3 METHODS

### 3.1 RF pulse design

All methods were used to design refocusing pulses (180° flip) with a slice‐thickness of 2 mm, maximum RF amplitude of B_1,max_ = 13 μT, maximum gradient slew rate of S_max_ = 200 $mTm^{-1}ms^{-1}$ and G_max_ = 40 $mTm^{-1}$. Time bandwidth products (TBP) 2 and 4 were used, and the number of slices “N” was varied from 2 to 12. For each N, we designed 1 set of pulses with a fixed slice‐separation of 14 slices (i.e., 28 mm from center to center) and 1 set with a fixed FOV of 200 mm, so a slice‐separation of $\frac{200mm}{2mm}\frac{1}{N}$ slices. All pulse designs were implemented in MATLAB 2015b (The MathWorks, Natick, MA), each pulse starting with 2048 samples and a sufficiently high sampling rate to avoid aliasing and numerical inaccuracies at high frequencies.

All the linear phase examples studied in this work started with the same Shinnar‐Le Roux (SLR)‐designed SB pulse. The SLR refocusing (assuming crusher gradients) pulse was designed using a finite impulse response (FIR) filter design approach, solved using a convex optimization approach, adapted from Sharma et al.^15^ In‐ and out‐of‐slice ripples were set to 1%. MBv and vMB pulses were designed as described above. MB modulation was completed using a phase‐optimized scheme as in Wong.^11^ Optimal phase‐offsets were obtained using MATLAB’s fmincon function—these were always the same for a given *N*, and so phase‐offsets were not adapted to match individual pulse designs.

Non‐linear MBv pulses were root‐flipped pulses, which were designed as described in Sharma et al.^15^ with publicly available code (https://www.vuiis.vanderbilt.edu/~grissowa/software.html). For this work, the ripple relations were set to design a single refocusing pulse instead of a matched‐excitation as originally proposed. Moreover, the Monte Carlo search for optimal root‐patterns was replaced by a genetic algorithm as implemented in MATLAB 2015b, which we previously found to give slightly improved results.^30^

Non‐linear vMB were chosen as quadratic phase pulses, which were designed by first designing a minimum phase pulse with the same slice characteristics as the linear phase pulse. The minimum phase pulse was reduced in RF power by evaluating its equivalent Cayley‐Klein β representation^32^ and inverting all its β‐roots on the bottom half of the unit circle, as described in Shinnar.^31^

PINS^16^ pulses were designed by appropriately undersampling the same linear phase SB waveform depending on the ratio of slice‐thickness to slice‐separation. Code to produce such pulses was based on source files downloaded from https://bitbucket.org/wgrissom/lowpeakpowermbrf/overview. PINS RF blips were made as short as possible to minimize pulse duration, putting it in line with other time‐optimal approaches in this study. Therefore, per PINS pulse, RF blips varied in duration (as dictated by B_1,max_) but gradient blip duration was fixed (as limited by gradient slew‐rate). MultiPINS pulses were designed by first designing a PINS pulse and then adding a reshaped MB pulse using a mixing ratio “M,” defined in Eichner et al.^17^ as

(5) $$RF_{\mathrm{MultiPINS}}=MRF_{\mathrm{MB}}+\left(1-M\right)RF_{\mathrm{PINS}}$$

The mixing‐ratio was increased from 0 to 1 (in steps of 0.005) to minimize pulse duration without exceeding B_1,max_ (see Supporting Information Figure S1). For each value of M in Equation (5), $RF_{\mathrm{PINS}}$ was designed as described earlier. To design $RF_{\mathrm{MB}}$, the same singleband waveform used for $RF_{\mathrm{PINS}}$ was multiplied by a modulation function without phase‐optimization. Subsequently $RF_{\mathrm{MB}}$, as defined for a constant gradient, was reshaped for the blipped PINS gradient using a VERSE algorithm, as described by Equation 8 in Eichner et al.^17^ For time‐optimal PINS and MultiPINS, better performance can be achieved with short sampling times thanks to shorter RF blips. In this work, the sampling time was set to 1.21 μs for all time bandwidth product 2 designs and 3.37 μs for all time bandwidth product 4 designs.

### 3.2 Evaluation of gradient distortion

**Figure 2:**
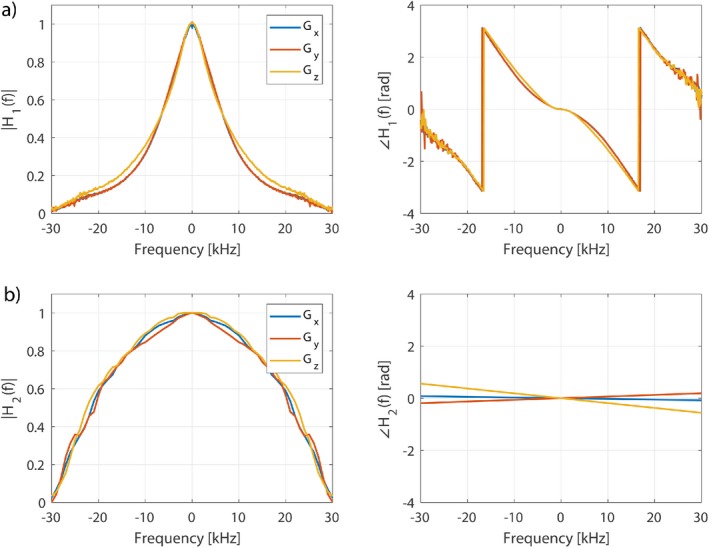
(a) Measured impulse response function h_1_ in the frequency domain for all gradient axes at frequency resolution 156 Hz. The x‐ and y‐axes are very similar, while the z performance is slightly different. (b) h_2_ based on a published measurement from a different vendor, reconstructed with a frequency resolution of 1 kHz. The phase profile on the right is estimated to be linear (i.e., constant time‐delay for all frequencies) for simplicity. Please note that although h_1_ is a true experimental measurement, h_2_ is only an approximation reconstructed from Testud^34^

In this work, we use 2 different GIRFs $h_{1}\left(t\right)$ and $h_{2}\left(t\right)$ that are related to scanners from 2 different manufacturers, corresponding to a Philips Achieva 3T (Philips Healthcare, Best, The Netherlands) and a Siemens Magnetom 3T (Siemens Healthcare GmbH, Erlangen, Germany), respectively. $h_{1}\left(t\right)$ was measured experimentally using an image‐based procedure similar to that reported in Papadakis et al.,^33^ and $h_{2}\left(t\right)$ was reconstructed manually from Testud.^34^ The 2 frequency responses are shown in Figure 2 and are quite different. Please note, however, that although $h_{1}\left(t\right)$ was measured experimentally, $h_{2}\left(t\right)$ should only be treated as an approximation. Both GIRFs included measurements for all 3 gradient axes, but in this work we used only the z‐axis gradient coils (i.e., exciting purely transverse slices). Unless specified, $h_{1}$ was used for the results presented in this article.

For each candidate pulse design, the predicted gradient after distortion G_actual_(t) can be computed from the target waveform G_target_(t) by convolution:

(6) $$G_{\mathrm{actual}}\left(t\right)=G_{\mathrm{target}}\left(t\right)*h\left(t\right)$$

In practice, the convolution was computed using frequency domain multiplication, and the GIRF was linearly interpolated beforehand to account for any differences in frequency resolution. Bloch equation simulations (using Cayley‐Klein representation) were then performed using G_target_(t) and G_actual_(t) to find the target and predicted distorted slice profiles, respectively. Slice profiles were represented using flip angles $\theta \left(z\right)=arccos\left(M_{z}\left(z\right)\right)$, and the normalized RMS error (NRMSE) was computed between the 2 profiles and normalized to the target profile. Flip angle representation was chosen to make our analysis independent of the final use of these pulses. A specific measure relevant to spin‐echo refocusing is the $\beta^{2}$ profile from the Cayley‐Klein parameters, which was also calculated along with the phase deviation for $\beta^{2}$ and flip‐angle profiles. Slice profile error was computed for both the FOV of a single pack of slices and 3 times this FOV. This distinguishes between distortions inside and outside the FOV being imaged, as the former relates to slice distortions leading primarily to blurred images, and the latter leads to residual ghosting and saturation effects. These errors are referred to as $\epsilon_{\mathrm{inside}}$ and $\epsilon_{\mathrm{outside}}$ respectively. Phase errors were also evaluated. Because phase is not well‐defined when simulating a 180° pulse, we quantified the through‐slice phase distortion for the pulses when scaled down to ~45° flip angle and also considered the phase profiles of $\beta^{2}$ without rescaling. In both cases, linear phase rolls common to all slices were discounted, because these could be balanced by appropriate rewinders/crushers. Finally, RF pulses with time‐variable gradients are known to suffer more from off‐resonance effects. To investigate this, off‐resonance simulations were conducted for an outer slice of an MB4 TB4 example at off‐resonance frequencies from 0 to 200 Hz.

### 3.3 Experimental validation

Slice profile measurements were performed on Philips Achieva 3T system (Philips Healthcare, Best, the Netherlands) whose frequency response is close to $h_{1}\left(t\right)$. Phantom experiments used a cylindrical phantom containing 100 mL of saline (9 g/L) doped with 1% gadolinium contrast agent (0.5 mmol/L Gd‐DOTA, Dotarem, Guerbet LLC, Bloomington, IN). RF pulses used were designed, based on a vendor SB waveform, as MB3 TB4.4 180° refocusing pulses, slice‐thickness 2 mm, center‐to‐center gap of 20 mm, and optimized for the constraints B_1,max_ = 13 μT, G_max_ = 31 $\text{mT}m^{-1}$, S_max_ = 200 $\text{mT}m^{-1}ms^{-1}$. Both RF and gradient waveform were designed at a sufficiently short sampling time, before being downsampled to the MR system sampling time of 6.4μs. To visualize the slice profile from these RF pulses in isolation, the pulses were scaled down by a factor of 3 (flip angle ~60°) and then incorporated into a 2D gradient‐echo sequence (TR = 500 ms, TE = 25 ms, 0.2 × 0.48 mm in‐plane resolution), with the read‐out gradient moved to the same direction as the slice‐selection gradient. Optimal phase‐offsets were chosen to produce real‐valued RF pulses (i.e. not complex‐valued), which could be described using purely signed AM.^30^ This was done to circumvent an additional known hardware issue with faithfully reproducing rapidly varying FM waveforms. This issue also led us to choose linear vMB rather than non‐linear vMB pulses, because the starting SB pulse in the non‐linear case is not real‐valued.

In vivo imaging was conducted on the same MR system, using a single healthy volunteer (male, 27 y) after the sequence, and the study was approved by our local ethics board. The same RF and gradient designs as those from the phantom experiment were used, with exception that the original 180° RF refocusing pulses were scaled down by a factor of 6 so that they could be used as low‐tip excitation pulses. A gradient‐echo sequence (TR = 100 ms, TE = 14 ms, slice‐thickness 2 mm, 0.75 × 0.6 mm in‐plane resolution) with a blipped‐CAPI shift acquisition scheme was used,^35^ and MB data were reconstructed with a SENSE‐based algorithm using ReconFrame (GyroTools GmbH, Zurich, Switzerland).

Code to reproduce such VERSE and PINS RF and gradient pulses (and to perform the related simulations) has been made publically available on our GitHub repository (https://github.com/mriphysics/verse-mb).

## 4 RESULTS

**Figure 3:**
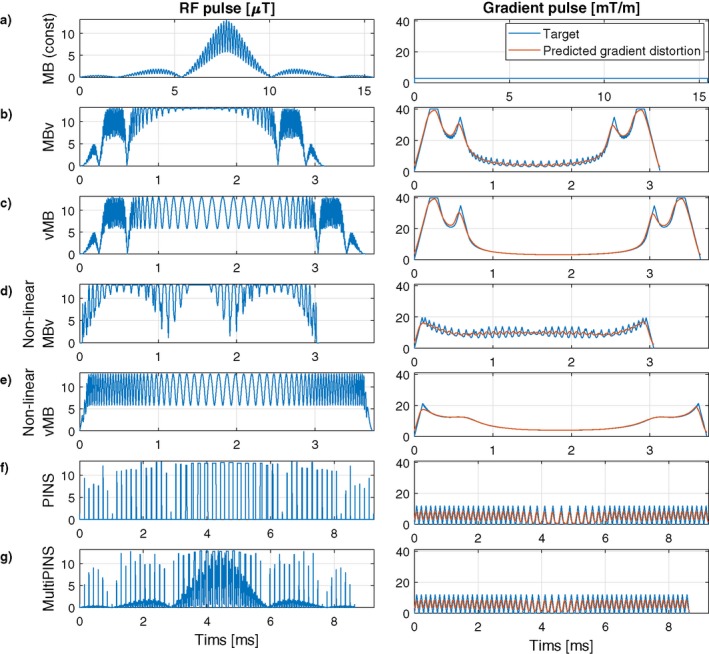
Example RF and gradient waveforms for every technique used in this work for an MB3, time bandwidth product 4 design with 2 mm slices and 28 mm slice‐gap. The 2 columns display RF (only the modulus is shown for simplicity) and gradient waveforms, respectively, on different time scales. The effect of gradient distortion from GIRF h_1_ is shown in orange. (a) Linear phase constant gradient MB pulse. (b) Multiband modulation followed by VERSE (MBv), linear phase. (c) VERSE followed by multiband modulation (vMB), linear phase. (d) MBv for non‐linear phase. (e) vMB for non‐linear phase. (f) PINS. (g) MultiPINS. The impact of the gradient distortion is shown in Figure 4

**Figure 4:**
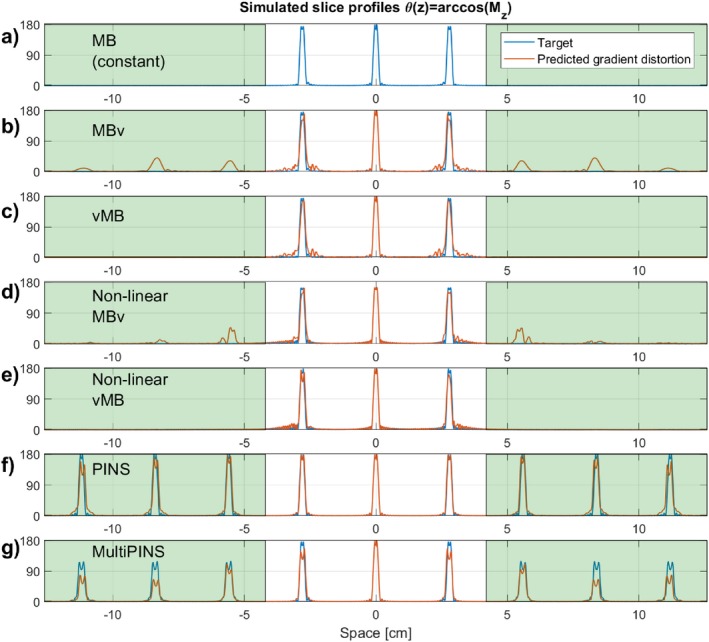
Slice profile distortions for the MB3, time bandwidth 4 refocusing pulses shown in Figure 3. Slice profiles are shown in flip‐angle representation. For VERSE methods, the gradient distortions seen in Figures 3b–3e lead to distortion within the imaging slice‐pack, as well as the additional excitation of ghost slices outside the imaging FOV for MBv methods. For PINS methods, gradient distortion leads to slice distortion within and outside the FOV, but often the error outside the FOV is ignored. The shaded and unshaded region shows where ϵ*_outside_* and ϵ*_inside_* are defined. A quantitative analysis of these errors across different designs is shown in Figure 5

Figure 3 shows the temporal profiles of MB3 RF and target gradient pulses (G_target_), as well as the predicted distorted gradient (G_actual_) assuming GIRF $h_{1}\left(t\right)$. Application of VERSE leads to a compression of the RF waveforms with the MBv methods (Figures 3b, d) showing the smallest durations for this design, with little difference between linear and non‐linear phase. The gradient waveforms from Figures 3b, d show that when VERSE is performed on the MB pulses, the resulting gradient waveforms have high temporal bandwidth. The slice profile simulations in Figure 4 show that these designs result in artifacts at ghost‐slice locations when the effect of limited gradient‐system bandwidth is included. For PINS pulses, although temporal gradient distortion is relatively severe, because RF and gradients are not usually active at the same time, the effect of distortion as shown in Figure 4f is relatively minor. This is not the case for MultiPINS (Figure 4g) because RF and gradients are active simultaneously.

**Figure 5:**
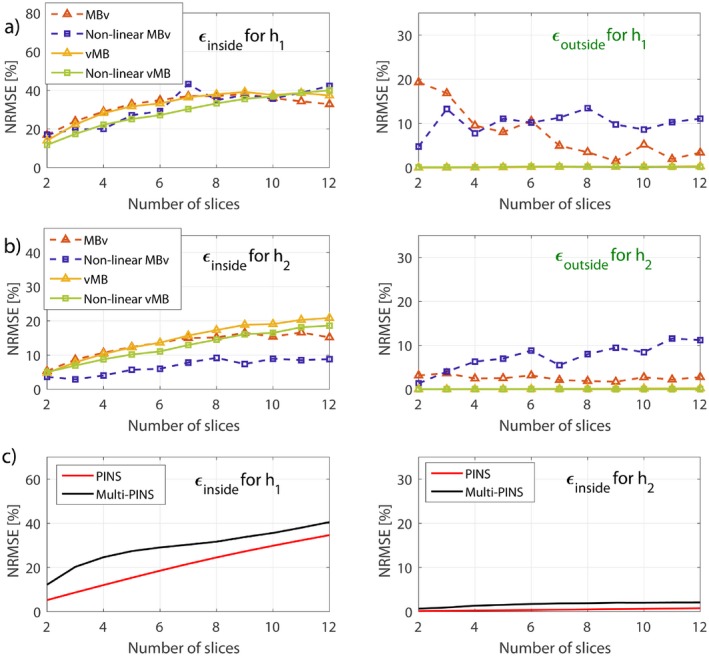
Slice profile error for the different techniques investigated inside and outside an imaging slice‐pack (as illustrated in Figure 4). (a) Results found for a measured GIRF h_1_. The error induced by finite temporal bandwidth of gradient systems is fairly consistent within the slice‐pack (left‐plot), but outside the slice‐pack there is negligible error from vMB methods. (b) Same principle for a second GIRF h_2_ with higher temporal bandwidth. (c) PINS and MultiPINS results in less slice profile error than VERSE methods. When temporal bandwidth is increased to h_2_, PINS methods benefit greatly. ϵ*_outside_* is not shown here as it is irrelevant in practice. These results are for the set of pulses calculated for a fixed slice‐separation of 28 mm. A similar plot for a fixed FOV is shown in Supporting Information Figure S2, and an analysis for refocusing profiles is shown in Supporting Information Figure S3. Further analysis of distortion of through‐slice phase coherence is presented in Supporting Information Figure S4 and S5, for flip‐angle and refocusing profiles, respectively

Figure 5 compares the slice profile errors inside ($\epsilon_{\mathrm{inside}}$) and outside ($\epsilon_{\mathrm{outside}}$) the imaging slice‐pack for various different N as predicted by both GIRFs ($h_{1}$ and $h_{2}$). As expected, the lower bandwidth GIRF shows greater distortion. All methods have some error within the FOV—this is also visible on Figure 4 and is mainly attributed to slice profile distortion and localized ringing. The MBv methods (linear and non‐linear phase) are noticeably more susceptible to error outside the FOV—this corresponds to the ghost slices that are excited because of distortion of gradient pulses with high temporal bandwidth. This is absent in the vMB methods, demonstrating the benefit of this approach. Supporting Information Figure S2 shows similar results for the case of fixed field‐of‐view, and Supporting Information Figure S3 shows such results when considering spin‐echo refocusing profiles ($\beta^{2})$ instead of flip‐angle representation. In Supporting Information Figure S3, it can be seen that $\epsilon_{\mathrm{outside}}$ decreases for MBv and PINS methods, but the relations between all methods remain the same. Supporting Figures S4 and S5 show the additional average phase deviation across the slice profile because of gradient distortion, which was found to be of 1–5${}^{\circ }$ additional loss in phase coherence.

**Figure 6:**
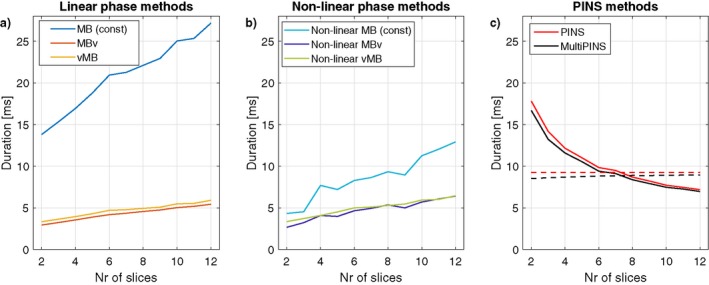
RF pulse durations associated with each method (split in (a) linear phase, (b) non‐linear phase, and (c) PINS designs) as a function of N, for TBP = 4, maximum G_max_ = 40 mT m^−1^, and B_1,max_ = 13 μT. All results are shown for the case of a fixed imaging FOV, except for the dashed lines in (c) that are for a fixed slice‐separation of 14 slice‐thicknesses for every N. vMB methods are only slightly longer in duration than the MBv methods (10.7% and 9.1% on average for linear and non‐linear phase, respectively). Depending on the ratio of slice‐separation to slice‐thickness, they can be more time‐efficient than PINS and MultiPINS pulses. Supporting Information Figure S6 shows a similar figure for TBP = 2

The primary objective for our designs was to produce time‐optimal RF pulses. Figure 6 shows the pulse durations for the proposed vMB and MBv methods, alongside existing methods, for designs with a fixed FOV of 200 mm and therefore varying relative slice‐separation with N. The figure shows that VERSE can be used to drastically reduce the duration of the original linear phase constant gradient RF pulse by around a factor of 5. vMB is only slightly less time‐efficient than MBv (10.7%) even though the former has been shown to suffer from fewer slice profile distortion effects. Similarly, non‐linear vMB was on average 9.3% longer than non‐linear MBv. Duration of PINS and MultiPINS is invariant with respect to N, but varies with slice‐separation. To highlight this, Figure 6c shows the case for variable separation as described, but also for variable N with fixed separation (dashed lines). The other (non‐PINS) methods are not as sensitive to changes in slice‐separation.

**Figure 7:**
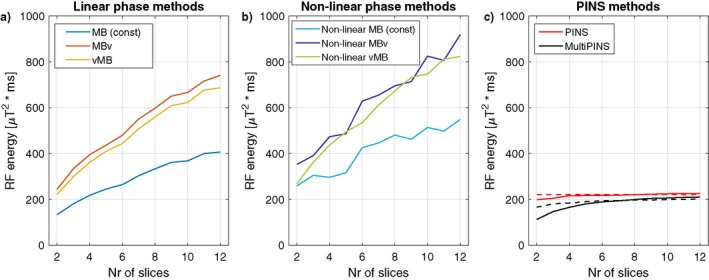
RF energy (units $\mu T^{2}\text{ms}$, proportional to energy) as a function of the number of slices excited for the case of fixed FOV of 200 mm for (a) linear phase pulses, (b) non‐linear phase pulses, and (c) PINS methods. Results for the case of fixed slice‐separation is shown in dashed lines. An equivalent version for TBP = 2 pulses is available in Supporting Information Figure S7

Figure 7 shows RF energy associated with each of the methods, calculated by integrating the square amplitude of each pulse (units are $\mu T^{2}\text{ms}$ which is proportional to the energy). The graph shows a reciprocal relation to pulse duration. Supporting Figures S6 and S7 show similar results for the case of time bandwidth product 2.

**Figure 8:**
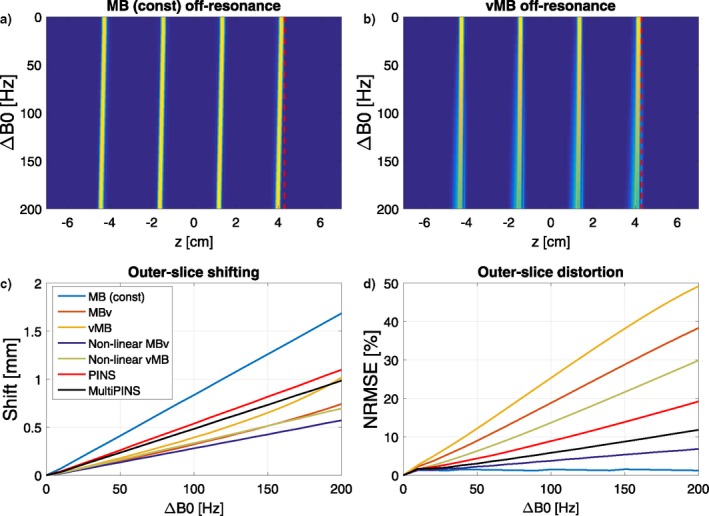
Off‐resonance simulations for the MB4 pulses, shown for the (a) constant gradient MB and (b) vMB case, with a vertical dashed line as a visual cue. Constant gradient MB pulses experience more slice‐shifting than vMB pulses because of their longer durations, but vMB suffers more from slice distortion at off‐resonance frequencies. Subfigure (c) shows how much the outer‐slice for each technique experiences a spatial shift as a function of frequency. This shows that longer pulses experience a larger shift. (d) Outer‐slice distortion as compared in NRMSE from its on‐resonant equivalent. MBv and vMB experience the worst off‐resonance distortion. A similar plot for TBP = 2 pulses is shown in Supporting Information Figure S8

Time‐variable gradients also lead to complex off‐resonance behaviour as shown by Figure 8. The top row of this image shows the simulated profile of a MB4 time bandwidth product 4 example, in a constant gradient MB case (Figure 8a) as well as a verse MB case (Figure 8b). Off‐resonance results in a shifting of the slices and a degradation of the slice profile. We quantitatively distinguish these 2 effects for all pulse design methods from this work by reporting the shift experienced by an outer‐slice of the MB4 pack in Figure 8c and the NRMSE for the degraded slice when corrected for their spatial displacement (found by maximal cross‐correlation) in Figure 8d. The latter method was also used in Eichner et al.^17^ The largest shifts are experienced by the longest pulses, as they spend more time off‐resonant. The linear phase vMB and MBv methods perform the least favorably in terms of slice distortion off‐resonance. The effect is less pronounced for lower time bandwidth (i.e., shorter) pulses (shown in Supporting Information Figure S8). The effect of gradient bandwidth‐related distortion on off‐resonance sensitivity was found to be insignificant.

**Figure 9:**
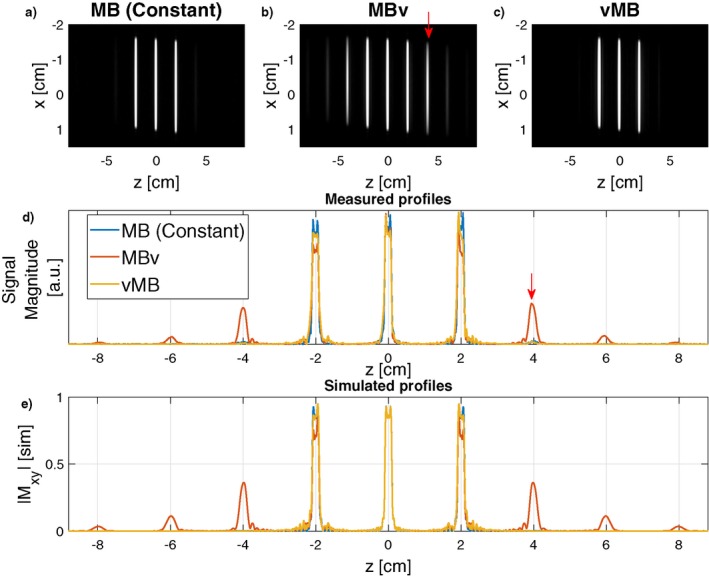
Slice profile measurements using RF and gradient pulses (*N* = 3, TBP = 4.4 refocusing pulse, slice‐thickness = 2 mm, gap = 20 mm). After VERSE, RF refocusing pulses were scaled down by a factor of 3 such that they could be used as excitation pulses. (a) Slice profile produced with a constant gradient shows very low artifact level. The MBv slice profile in (b) shows significant artifacts at well‐defined ghost locations at multiples of the slice‐gap outside the original FOV (red arrows in b and d), which would lead to image artifacts. The vMB method in (c) effectively avoids this problem. (d) More clearly illustrates the artifacts, which corresponds with the simulated predictions in (e)

Figure 9 shows experimentally measured slice profiles for an MB3 pulse for constant gradient, MBv, and vMB methods (linear phase). The relevant gradient system is characterized by $h_{1}$. Both MBv and vMB have some distortion in the outer slices (as expected from simulation, see Figure. 4) but the MBv method also has strong ghost slices (as indicated by the red arrows) that are not seen for vMB, again as expected. The pulses used in this experiment were also simulated using the predicted distorted gradient, and in Figure 9e are shown to resemble the measured results. Note that in this experimental validation, the difference in duration between linear MBv and vMB pulses was 30.9% which is greater than the average of 10.7% reported above. This is because MB pulses were constrained to have real‐valued (AM) modulation, and it was found that this constraint affects the performance of vMB much more than MBv.

**Figure 10:**
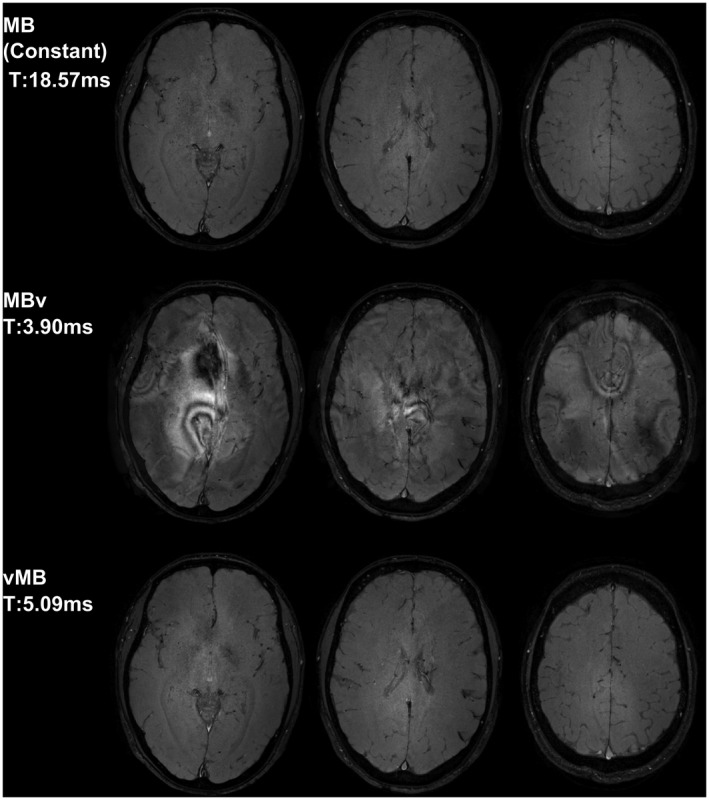
In‐vivo results for MB3 pulses (gradient‐echo, 2 mm slice‐thickness, 0.75 × 0.56 mm in‐plane FA = 30°, B_1_ = 2.17 μT, TE = 14 ms, TR = 100 ms, real‐valued RF pulses) using constant gradient (top), MBv (middle), and vMB pulses (bottom). MBv pulses excite regions outside the FOV, which introduce signal into other images, creating strong artifacts. The vMB pulses avoid this behavior and produce equivalent image quality as the constant gradient pulses, but with a 3.6× shorter pulse duration. The relative performance between MBv and vMB pulses (vMB pulse duration is 30% longer than MBv) is above the average (10.9%) partly because the design in this case was constrained for AM‐only modulation; this is required to avoid further artifacts on our system

Figure 10 shows in vivo gradient‐echo images acquired using a similar pulse. The ghost slices lead to significant reconstruction artifacts because of unresolved aliasing in the MBv case, which are avoided by using vMB pulses. Figure 9 shows that there is a small residual artifact at the ghost slice location (±4 mm), in both the constant gradient and vMB results, which was attributed to a residual RF chain instability that we could not correct for. Because the artifact is present in the standard constant gradient case, it can be assumed to be unrelated to gradient bandwidth artifacts, and as evidenced by Figure 10, this does not lead to an obvious imaging artifact.

## 5 DISCUSSION

In this work, we examined the effect of limited temporal bandwidth of gradient systems on the performance of multiband (MB) RF pulses with time‐variable gradients. We explored the use of VERSE to create short MB pulses and compared performance of using VERSE on MB pulses (called MBv) with VERSE on singleband (SB) pulses that are subsequently modulated to make them MB (called vMB). As hypothesized, the vMB method resulted in temporally smoother gradient waveforms with reduced distortion artifacts.

The general problem of gradient distortion is illustrated in Figures 3 and 4, showing the type of effect that would be expected from a gradient system characterized by impulse response function (GIRF) $h_{1}$ (shown Figure 2). Figure 9 shows some equivalent experimental measurements and simulated errors, confirming this prediction. Figure 10 shows the resulting errors in SMS image acquisition.

In general, gradient distortion leads to distortion of the individual slice profiles quantified by $\epsilon_{\mathrm{inside}}$ and excitation of “ghost slices” that tend to appear at multiples of the multiband slice locations (see Figures 4 and 9) quantified by $\epsilon_{\mathrm{outside}}$. Results (Figure 5 and Supporting Information Figures S2 and S3) show that $\epsilon_{\mathrm{inside}}$ is similar between MBv and vMB methods, but was much lower for the higher bandwidth gradient system characterized by $h_{2}$. This is to be expected because the individual slice profiles are related to the SB RF and gradient waveforms—these are affected in a similar way by both MBv and vMB approaches and are more strongly distorted by $h_{1}$ than $h_{2}$. The only anomaly for the $\epsilon_{\mathrm{inside}}$ results is the surprisingly good performance of non‐linear MBv (Figure 5b, purple trace) that we cannot explain.

MBv and vMB approaches differ in that MBv results in gradient waveforms that are modulated at the multiband modulation frequency—distortion therefore leads to “ghost slices” that are not present in the vMB method. Figure 5 shows that this is the case, and these unwanted slices can be seen in Figure 9. When these ghost slices fall within the anatomy they appear as unreconstructed artifacts, as in Figure 10. In this work, we used 180° refocusing pulses as a main example application, to allow comparison with other existing pulse design methods. In the refocusing case, the image artifacts seen would depend on the $\beta^{2}$ profile (Supporting Information Figure S3) as well as the excitation pulse used. Refocusing pulse errors outside the FOV would only lead to image artifacts if used in combination with an excitation pulse with a similar artifact problem. It would therefore be possible to avoid these artifacts by using excitation pulses with better performance, however, it should be expected that to obtain short echo times, excitation pulses may be designed with the same approach and would be likely to have similar ghosts. Excitation for short TR gradient echo sequences is another possible use of these pulses,^36^ and Figure 10 used this application as a demonstration, because it allows for a straightforward visualization without the need to design an additional excitation pulse.

In addition to magnitude errors, phase distortions were also investigated (Supporting Information Figures S4 and S5). In general, these errors were found to be small, at ~1–5^°^ in average phase‐deviation in both refocusing and flip‐angle profiles.

The MBv method studied here creates RF pulses that are similar in both RF and gradient waveform to those created by an optimal control method (see Figure 10a in Grissom et al.^18^). Pulses from the latter method are expected to be shorter in duration than those created by the MBv method, because RF and gradient are jointly optimized instead of being done sequentially. The resulting waveforms have similar temporal characteristics, so we expect the gradient bandwidth related errors to also be similar. In contrast, the proposed vMB method does not suffer from these effects because the gradient waveforms have an inherently lower temporal bandwidth.

Gradient‐related slice profile errors are more pronounced for gradient systems with lower temporal bandwidth, however, they are still expected to be present on gradient systems with a higher temporal bandwidth. Predictions made using a higher bandwidth GIRF $h_{2}$ constructed from published results on a system from an alternative vendor (Testud^34^; Figure 2) still show higher $\epsilon_{\mathrm{outside}}$ for the MBv methods, compared with vMB (Figure 5). In general, the variability of gradient system bandwidth that causes the reported differences in performance has not been problematic for MB methods in mainstream use, because these use constant selection gradients that are faithfully reproduced on all systems. A move to more rapidly varying gradient waveforms places more demand on the gradient system and can lead to the errors shown in this work. Aside from reducing errors, another advantage of the vMB approach is that cross‐platform performance would be expected to be more similar, which may be desirable for standardized protocols.

Previous work has also considered the gradient bandwidth‐related slice profile errors. In Hargreaves et al.,^19^ the gradient waveform after VERSE was low‐pass filtered up to 50 kHz to smooth out such effects. Another study showed how small mismatches between RF and gradient timings can lead to excitation errors.^37^ The solution proposed was to avoid RF and gradient amplitude being high simultaneously, which hampers the effectiveness of VERSE. RF characterization was not incorporated in this study, however, previous literature has identified that this can be problematic.^38^, ^39^

The penalty in terms of pulse duration is illustrated by Figure 6. The use of time‐variable gradients significantly reduces duration when compared with constant gradient pulses, in comparison the difference between vMB and MBv is relatively minor, with vMB being only 10.7% and 9.1% longer than MBv (linear and non‐linear phase, respectively). These designs are typically shorter in duration than PINS/MultiPINS pulses for lower N (fixed FOV)—duration of PINS type pulses is not explicitly dependent on N but falls as the ratio of slice‐separation to slice‐thickness falls. It is also apparent that once time‐variable gradient waveforms are used, there is no longer a big difference in duration between the linear and non‐linear phase designs. For example, before use of VERSE, the linear phase MB pulses are on average 252% longer than the non‐linear phase versions, however, after application of VERSE this difference drops to below 8% for both MBv or vMB variants. This is because VERSE is more effective at reducing durations for constant gradient RF shapes with regions of low‐and‐high amplitude RF lobes. A more comprehensive design approach such as Rund et al.^24^ could potentially outperform VERSE and increase the gap between linear and non‐linear phase designs.

As Figure 7 shows, another cost of producing very short duration pulses is increased RF energy. In this respect, the PINS‐related designs are more effective—this work focused on short duration, which inevitably leads to higher energy. The choice of which approach to take is application‐dependent.

A limitation of all VERSE‐based methods is that they can lead to poor off‐resonance performance. Our results (Figure 8) also show this to be the case, however, the significance of this error depends on the application (and whether fat suppression is applied, for example). It is also significantly less for low TBP pulses (see Supporting Information Figure S8).

Simulation and experiment (Figures 4 and 9) both suggest that low temporal gradient bandwidth also leads to additional ringing effects local to each slice, even with the vMB method. In previous work, we have shown that these errors can be effectively mitigated by using an iterative correction scheme^40^ with knowledge of the GIRF. In Supporting Information Figure S9, we show an experimental proof that the same method can correct the vMB method to reduce additional slice profile errors. The disadvantage from this method, however, is that the iterative correction requires knowledge of the scanner GIRF, must be computed online, and is potentially gradient‐axis‐dependent meaning that it may need to be recomputed if the slice orientation changes.

An additional benefit for vMB methods is that they are potentially simpler to implement. Phase‐offsets and peak amplitude of the MB modulation function that leads to time‐optimality are known beforehand. In practice, this means that for a combination of SB RF shape and slice‐thickness, one only needs to store a library of time‐optimal SB RF and gradient shapes constructed using VERSE (depending on number of slices and hardware limitations). The required modulation function to then produce an MB pulse (see Equation (2)) is easily calculated online.

As discussed previously, although we expect optimal control MB pulse designs such as in Rund et al.^23^ will outperform the MBv designs in this work, we would also expect them to suffer from similar slice profile errors because the gradient pulses have similarly high temporal bandwidth. Design approaches based on optimizing a SB RF pulse and gradient waveform based on some other method, before subsequent MB modulation, may be a useful area for future development.

## 6 CONCLUSION

We propose a novel method for designing time‐optimal multiband RF pulses that are less susceptible to distortion related to the finite temporal bandwidth of real‐world gradient systems. We assessed our work with a measured and reconstructed GIRF, based on 2 major vendors. We conclude that such pulses would benefit future SMS imaging applications.

## Supporting information


**FIGURE S1**MultiPINS pulses in this study were optimized for time‐optimality. As more MB is added to the pulse, the duration of the pulse decreases. The time‐optimal solution is found by maximizing M without violating the peak B1 amplitude constraint. This is a 1D version of Figure 2 found in (17).
**FIGURE S2**Slice profile error for the case of fixed FOV and flip‐angle represented slice profiles (compare with Figure 5).
**FIGURE S3**Slice profile error for the case of fixed slice‐separation of 28mm and using refocusing profiles (evaluated using β^2^ parameters). The error of ghost slices reduces, however the overall relationship between different methods remain the same. The same representation here was used to evaluate phase profile distortion in Supporting Figure S5.
**FIGURE S4**Phase profile deviation across slices for MBv, vMB, PINS and MultiPINS methods across the number of slices refocused. This figure shows the average phase error in the excited slices when the pulses are scaled to 45°. Linear phase rolls common to all slices were excluded. Therefore, the above results only show the increase in non‐linear phase deviation which cannot be corrected for using linear gradient fields. A 3° under‐tip is not a significant effect, even considering TSE sequences where CPMG conditions ought to be respected.
**FIGURE S5**Phase profile deviation across slices for MBv, vMB, PINS and MultiPINS methods across the number of slices refocused. This analysis is similar as shown in Supporting Figure S4 except the pulses were not rescaled, instead the phase corresponds to the phase of the β^2^ profile.
**FIGURE S6**RF pulse durations for fixed FOV, TBP = 2 as a function of the number of slices (compare with Figure 6, which was for TBP=4). The dashed lines in the graph for PINS methods show the durations for the case of fixed slice‐separation. The RF energy for these pulses are shown in Supporting Figure S7.
**FIGURE S7**RF energy vs Number of slices for fixed FOV and TBP = 2, corresponding to the pulses of Supporting Figure S5. The dashed lines in the graph for PINS methods show the RF energy for fixed slice‐separation. Compare with Figure 7, which was for TBP=4. As with Figure 7, the unit used here µT^2^ ms is proportional to the energy.
**FIGURE S8**Simulated slice‐shifting and slice‐distortion as a result of off‐resonance behaviour, as a function off‐resonance frequency ∆0. This is a TBP = 2 version of Figure 8. For VERSE pulses, off‐resonance effects are less damaging for lower TBP, making such pulses suitable candidates when spatial selectivity is less important.
