# Supplementary material for: Multiband RF pulse design for realistic gradient performance
Source: Magn Reson Med. 2018 Sep 14;81(1):362–76. doi: 10.1002/mrm.27411 (PMC6334175; doi:10.1002/mrm.27411)
Supplement: Supplementary file 1 — FIGURE S1MultiPINS pulses in this study were optimized for time‐optimality. As more MB is added to the pulse, the duration of the pulse decreases. The time‐optimal solution is found by maximizing M without violating the peak B1 amplitude constraint. This is a 1D version of Figure 2 found in (17). FIGURE S2Slice profile error for the case of fixed FOV and flip‐angle represented slice profiles (compare with Figure 5). FIGURE S3Slice profile error for the case of fixed slice‐separation of 28mm and using refocusing profiles (evaluated using β2 parameters). The error of ghost slices reduces, however the overall relationship between different methods remain the same. The same representation here was used to evaluate phase profile distortion in Supporting Figure S5. FIGURE S4Phase profile deviation across slices for MBv, vMB, PINS and MultiPINS methods across the number of slices refocused. This figure shows the average phase error in the excited slices when the pulses are scaled to 45°. Linear phase rolls common to all slices were excluded. Therefore, the above results only show the increase in non‐linear phase deviation which cannot be corrected for using linear gradient fields. A 3° under‐tip is not a significant effect, even considering TSE sequences where CPMG conditions ought to be respected. FIGURE S5Phase profile deviation across slices for MBv, vMB, PINS and MultiPINS methods across the number of slices refocused. This analysis is similar as shown in Supporting Figure S4 except the pulses were not rescaled, instead the phase corresponds to the phase of the β2 profile. FIGURE S6RF pulse durations for fixed FOV, TBP = 2 as a function of the number of slices (compare with Figure 6, which was for TBP=4). The dashed lines in the graph for PINS methods show the durations for the case of fixed slice‐separation. The RF energy for these pulses are shown in Supporting Figure S7. FIGURE S7RF energy vs Number of slices for fixed FOV and TBP = 2, corresponding to the pulses of [file MRM-81-362-s001.docx]

**
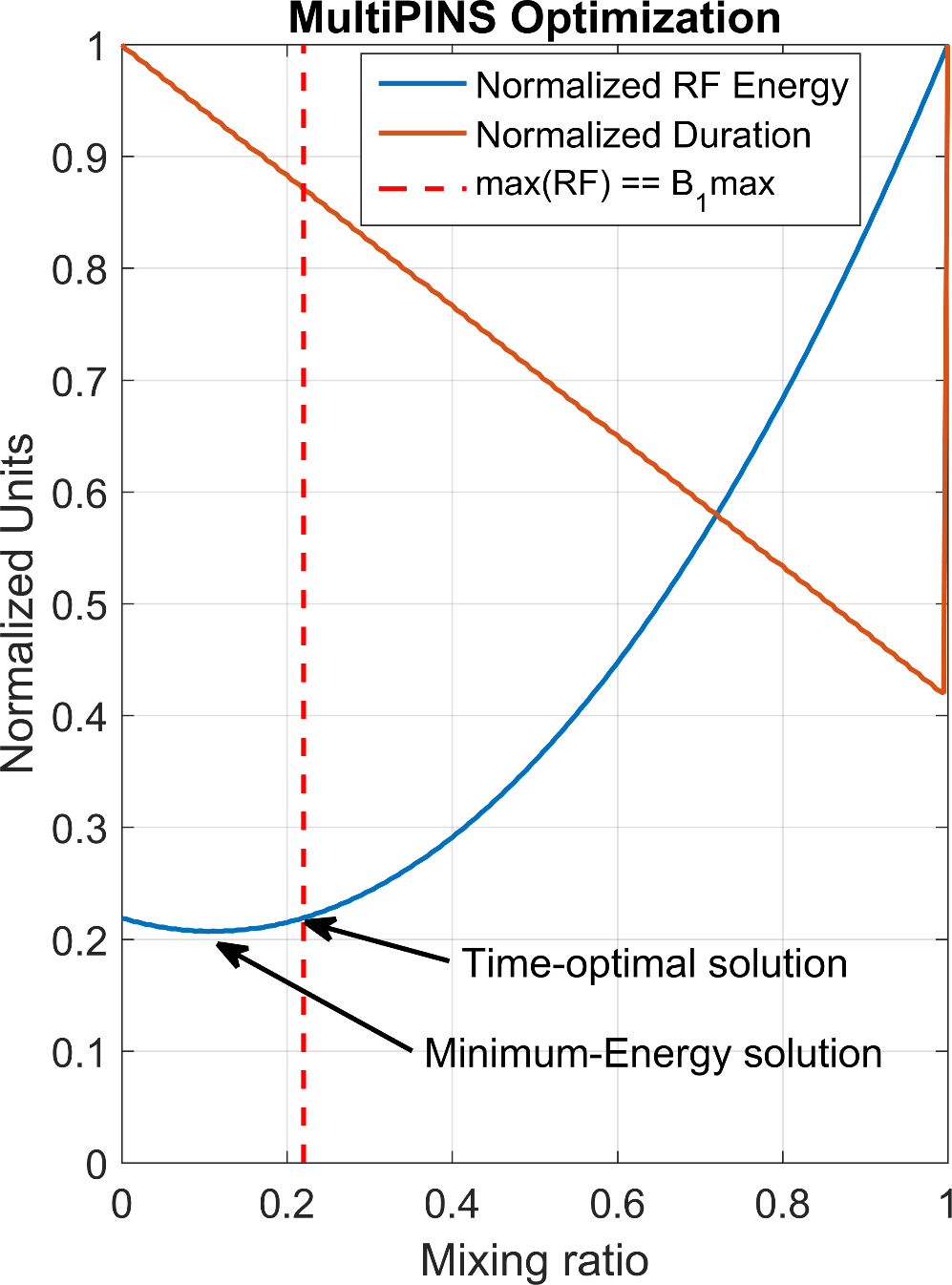
**

**Supporting Figure S1:** MultiPINS pulses in this study were optimized for time-optimality. As more MB is added to the pulse, the duration of the pulse decreases. The time-optimal solution is found by maximizing M without violating the peak B1 amplitude constraint. This is a 1D version of Figure 2 found in (17).


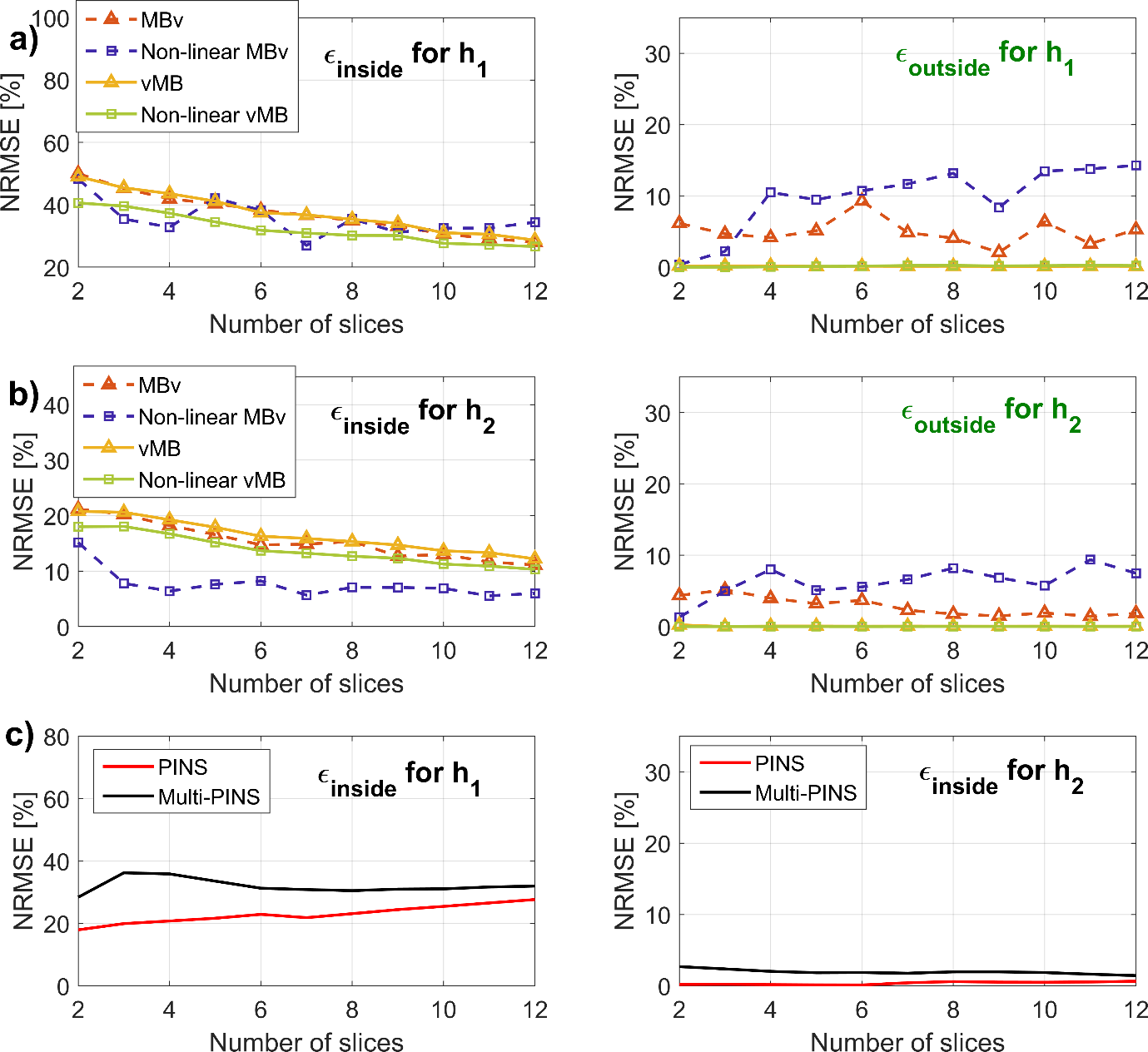


**Supporting Figure S2:** Slice profile error for the case of fixed FOV and flip-angle represented slice profiles (compare with Figure 5).

**
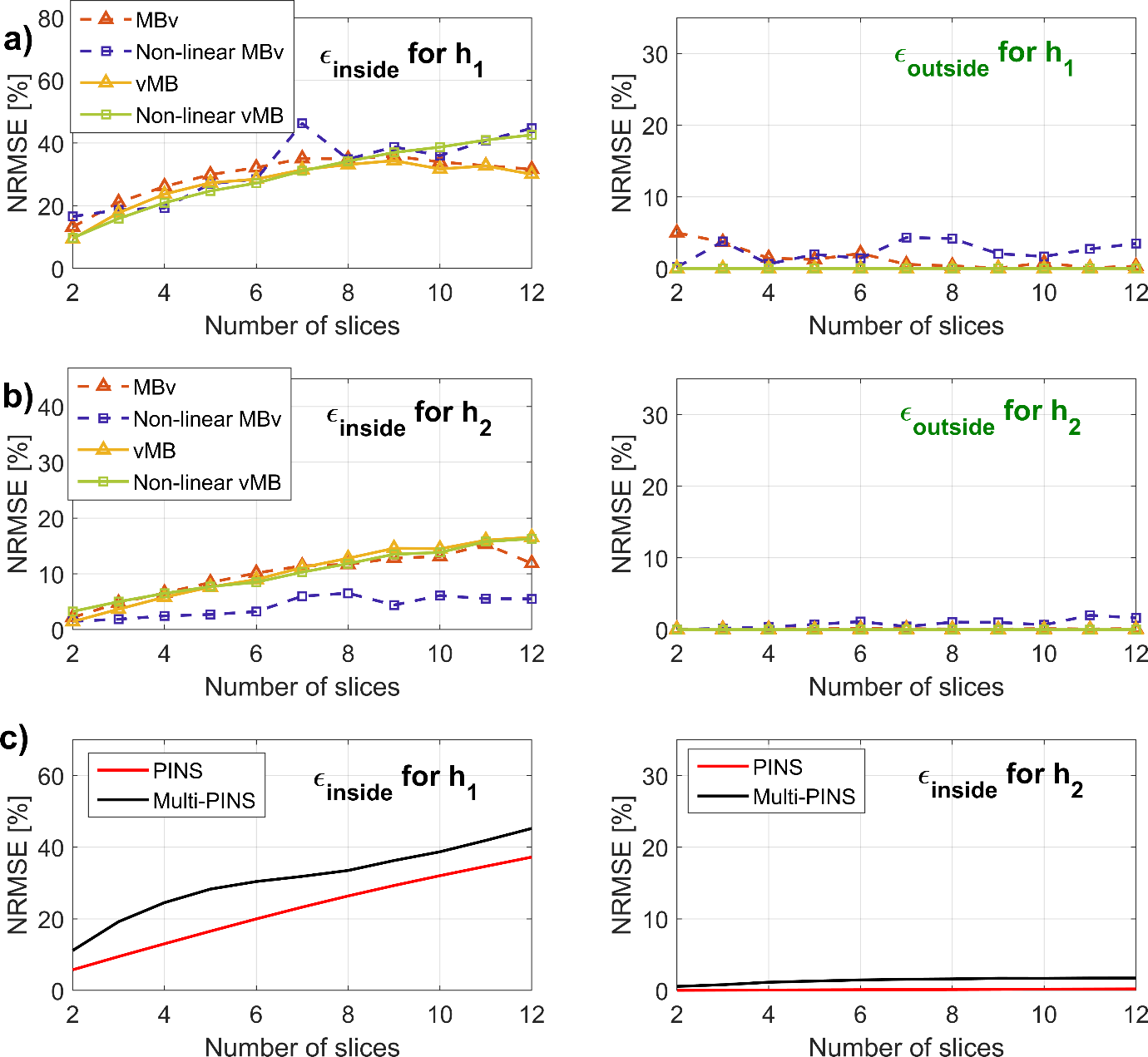
**

**Supporting Figure S3:** Slice profile error for the case of fixed slice-separation of 28mm and using refocusing profiles (evaluated using $\beta^{2}$ parameters). The error of ghost slices reduces, however the overall relationship between different methods remain the same. The same representation here was used to evaluate phase profile distortion in Supporting Figure S5.


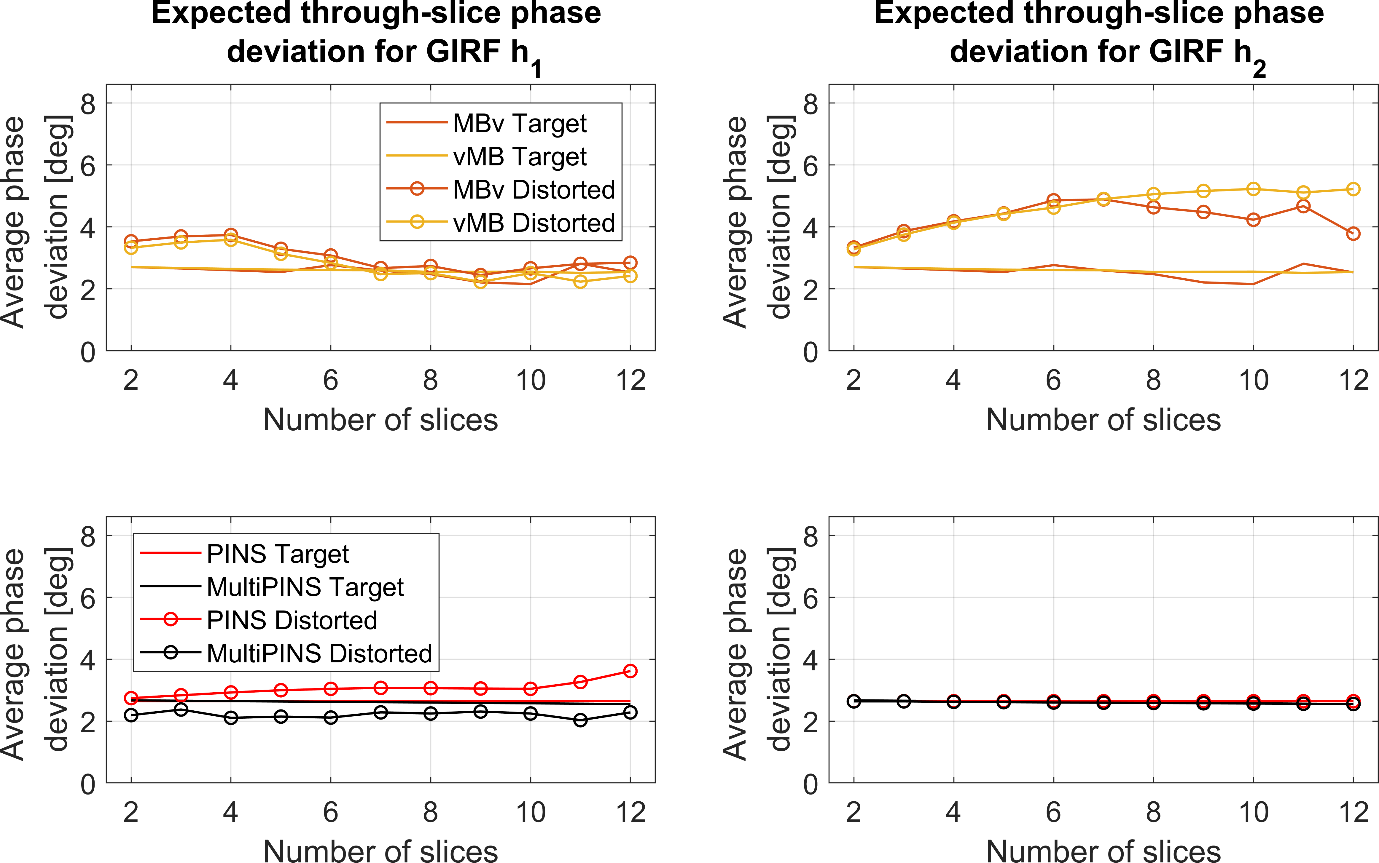


**Supporting Figure S4:** Phase profile deviation across slices for MBv, vMB, PINS and MultiPINS methods across the number of slices refocused. This figure shows the average phase error in the excited slices when the pulses are scaled to 45°. Linear phase rolls common to all slices were excluded. Therefore, the above results only show the increase in non-linear phase deviation which cannot be corrected for using linear gradient fields. A 3° under-tip is not a significant effect, even considering TSE sequences where CPMG conditions ought to be respected.


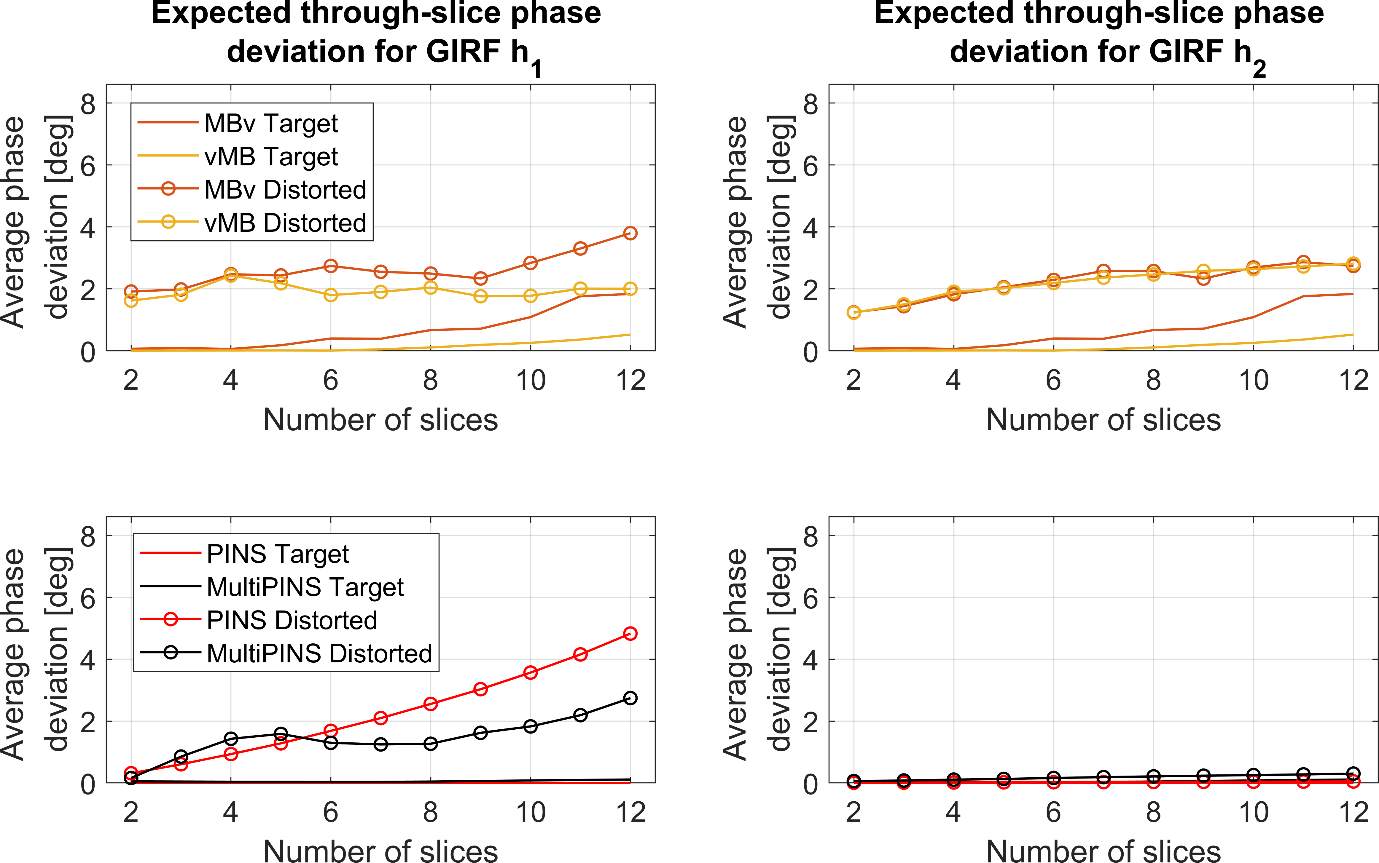


**Supporting Figure S5:** Phase profile deviation across slices for MBv, vMB, PINS and MultiPINS methods across the number of slices refocused. This analysis is similar as shown in Supporting Figure S4 except the pulses were not rescaled, instead the phase corresponds to the phase of the $\beta^{2}$ profile.


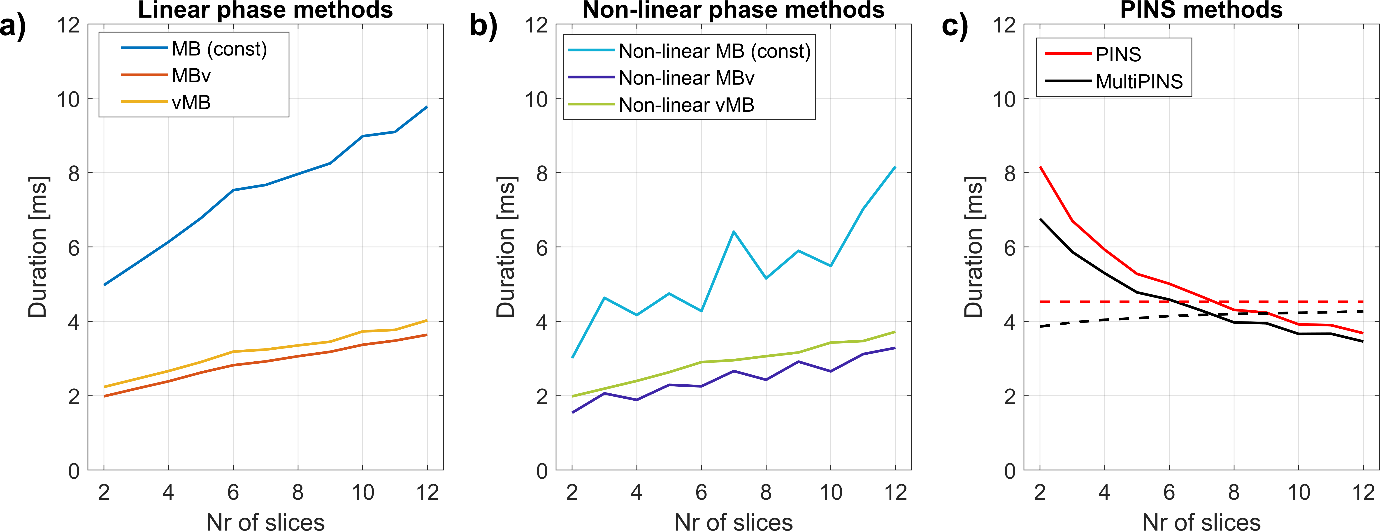


**Supporting Figure S6:** RF pulse durations for fixed FOV, TBP = 2 as a function of the number of slices (compare with Figure 6, which was for TBP=4). The dashed lines in the graph for PINS methods show the durations for the case of fixed slice-separation. The RF energy for these pulses are shown in Supporting Figure S7.


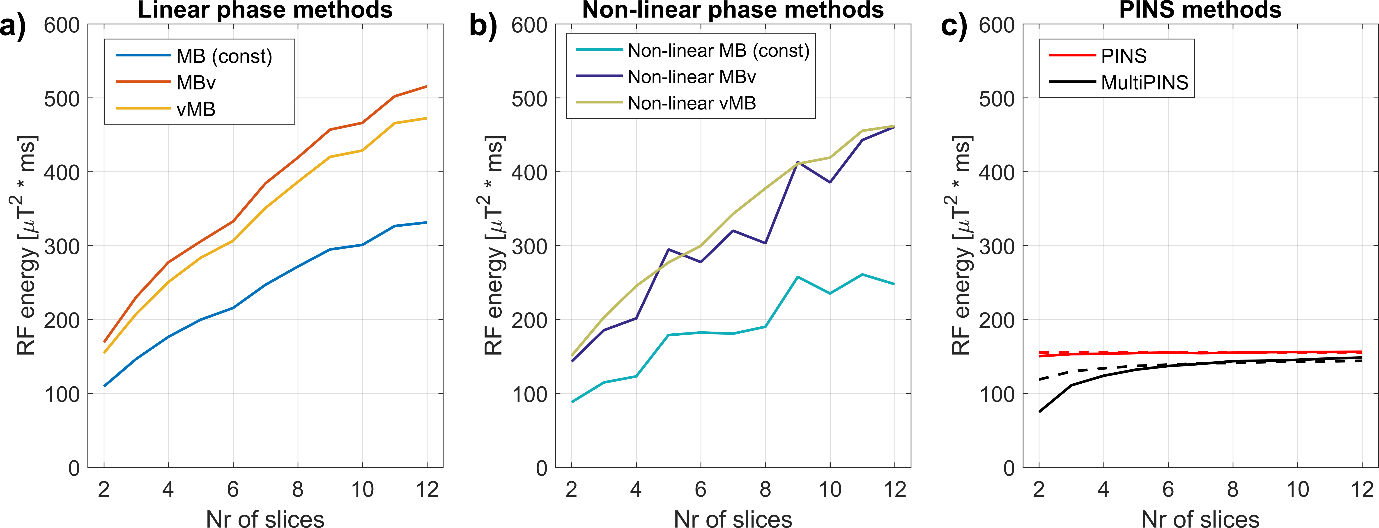


**Supporting Figure S7:** RF energy vs Number of slices for fixed FOV and TBP = 2, corresponding to the pulses of Supporting Figure S5. The dashed lines in the graph for PINS methods show the RF energy for fixed slice-separation. Compare with Figure 7, which was for TBP=4. As with Figure 7, the unit used here $\mu T^{2}\mathrm{ms}$ is proportional to the energy.


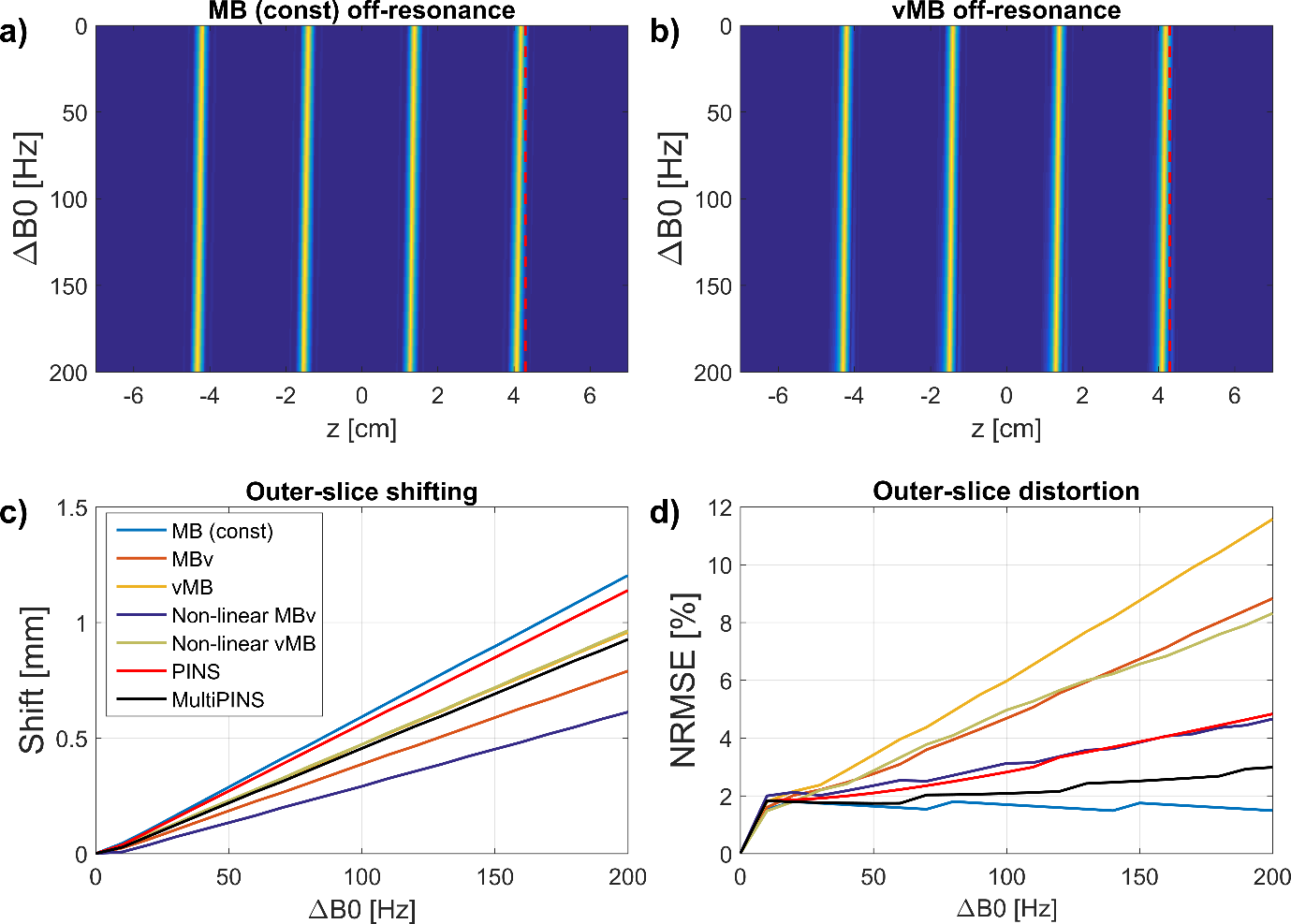


**Supporting Figure S8:** Simulated slice-shifting and slice-distortion as a result of off-resonance behaviour, as a function off-resonance frequency $\Delta B0.$ This is a TBP = 2 version of Figure 8. For VERSE pulses, off-resonance effects are less damaging for lower TBP, making such pulses suitable candidates when spatial selectivity is less important.

**
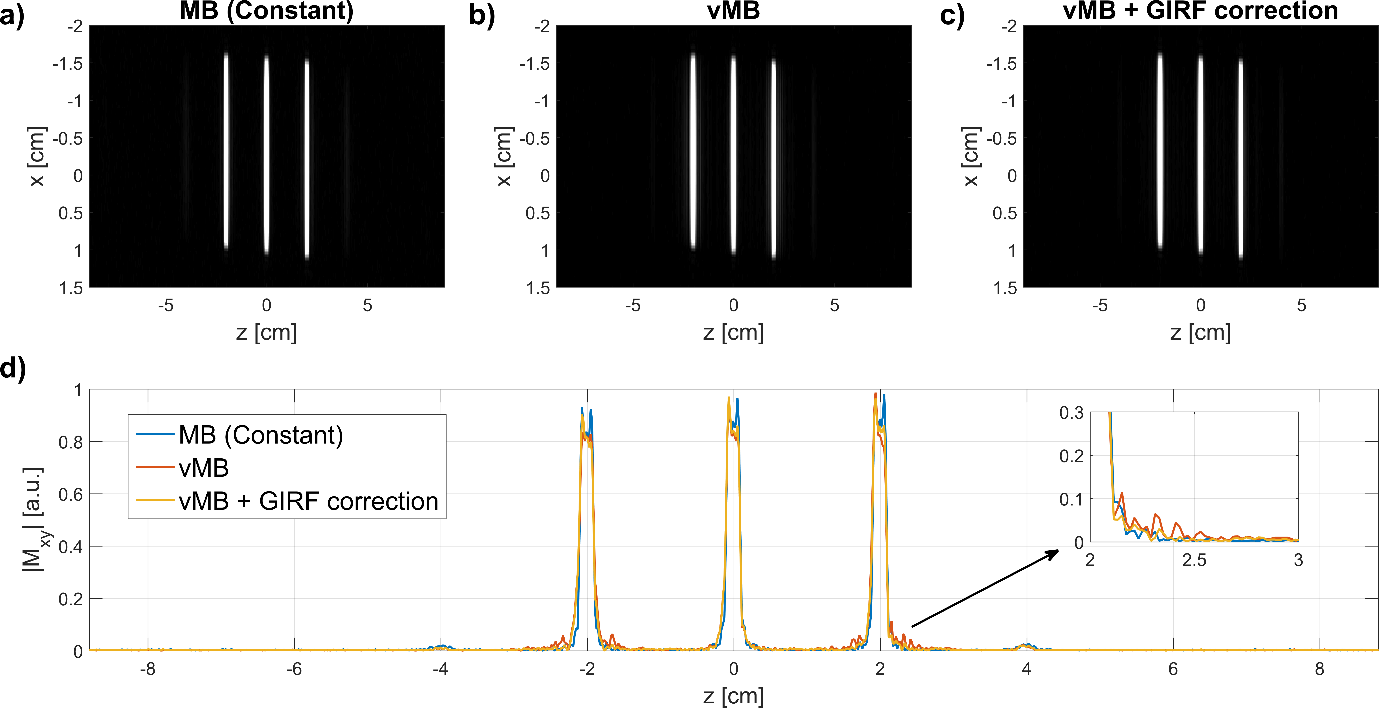
**

**Supporting Figure S9:** Experimental results showing how remaining slice distortions for the vMB method can be improved if the system GIRF is known. The same sequence as Figure 9 was used. **a)** shows a measured slice profile for an MB3 RF pulse with a constant gradient. **b)** shows the MB3 profile from a vMB pulse without GIRF-correction. **c)** shows the improvement achieved when the vMB RF pulse is iteratively corrected using the technique described in (41). **d)** more accurately shows the slightly improved passband and decreased sidelobes in the GIRF-corrected case. When used in-vivo, no clear benefit was visible when using vMB with GIRF correction, as such sidelobes barely affect imaging.
